# Structural Elucidation of a Novel Pectic Polysaccharide from *Zizyphus jujuba* cv. *Muzao*, a Potential Natural Stabiliser

**DOI:** 10.3390/plants15010059

**Published:** 2025-12-24

**Authors:** Zheng Ye, Wenjing Wang, Yumei Li, Chun Yang, Kai Mao

**Affiliations:** 1 Shanxi Institute for Functional Food, Shanxi Agricultural University, Taiyuan 030001, China; yezheng@sxau.edu.cn (Z.Y.); yangchungny@sxau.edu.cn (C.Y.); 2 College of Food Science and Engineering, Shanxi Agricultural University, Jinzhong 030801, China; 18155846901@163.com (W.W.); 15682681908@163.com (Y.L.)

**Keywords:** *Zizyphus jujuba* cv. Muzao, rhamnogalacturonan-I, structural characterization, hydrocolloid, highly branched polysaccharide, monosaccharide composition

## Abstract

The pH of fruit and vegetable juices is usually around 4.0. To adapt to the pH of fruit and vegetable juices, we developed a highly branched pectin as a natural stabiliser, whose polarity is well suited to conditions under weakly acidic conditions. The pectin content of jujube is high (about 2.0%), in which the polysaccharide content of Muzao (2.0–4.8%) is generally higher than the average value of the jujube. To separate the weak polar pectin in jujube, we extracted the crude polysaccharide (ZMP) with 4 times the volume of alcohol. Then we used Diethylaminoethyl (DEAE) cellulose (DEAE-52) ion-exchange chromatography to separate ZMP, and selected the fraction eluted with 0.2 M NaCl for gel purification to obtain ZMP2. After the hydrolysis of ZMP2 with TFA, four fractions, namely ZMP2n5, ZMP2y5, ZMP2n1, and ZMP2y1, were obtained. The purity, molecular weight, and monosaccharide composition of the above four fractions were determined. It was found that each fraction of ZMP2 contained large amounts of galacturonic acid (GalA) and glucuronic acid (GlcA), indicating that ZMP2 was most likely pectin, making it the natural, polar stabiliser we sought. To further determine the primary structure of ZMP2, we also performed FT-IR spectroscopy; methylation; one-dimensional mapping, including Proton Nuclear Magnetic Resonance (^1^H NMR), Carbon-13 Nuclear Magnetic Resonance (^13^C NMR) and Distortionless Enhancement by Polarization Transfer 135 (DEPT 135); and two-dimensional mapping, including Correlation Spectroscopy (1H-1H COSY), Heteronuclear Single Quantum Coherence (HSQC), Heteronuclear Multiple-Bond Correlation (HMBC), and Nuclear Overhauser Effect Spectroscopy (NOESY). In summary, the primary structure of ZMP2 should be as follows: the main chain is connected as →4)-α-D-GalAp-(1→3)-β-D-Galp-(1→, while the end glycosidic bonds of α-D-Galp-(1→ and α-L-Araf-(1→5)-α-L-Araf-(1→ are attached to the main chain by O-3 and O-6 bonds from →3,4)-α-D-GalAp-(1→ and →3,6)-β-D-GalAp-(1→, respectively.

## 1. Introduction

*Zizyphus jujuba* cv. *Muzao*, a primary jujube species cultivated in Shanxi Province, China, faces a significant economic challenge. Despite an annual output of approximately 50 million kilograms in Lin County, its profit potential is low due to low market prices for both fresh (0.4–0.6 yuan/500 g) and dried fruit (0.2–1.0 yuan/500 g) [1,2]. To enhance the value of this abundant agricultural resource, its in-depth valorization is imperative. Muzao fruit is known to be rich in polysaccharides (2.80–4.80% of dry matter), predominantly pectin [3,4]. Our previous work demonstrated that Muzao powder can function as a fortifying and hydrocolloid ingredient in fruit juice, a property attributed to its high pectin content [5,6]. This suggests that Muzao pectin could be a promising candidate for value-added product development. This aligns with the modern food industry’s growing consumer demand for “clean-label” products [7], driving the search for natural, plant-based hydrocolloids to replace synthetic stabilisers and thickeners [8,9,10].

Pectin is one of the most widely used natural hydrocolloids [11], particularly in acidic beverages like fruit juices [12]. However, the functionality of conventional commercial pectin, which is predominantly high-methoxyl homogalacturonan (HGA), is highly dependent on sugar content and acidity for gelation [13]. This has spurred significant interest in pectin fractions with distinct structural features, such as rhamnogalacturonan-I (RG-I) [14]. Recent studies have highlighted that RG-I-rich pectins can provide excellent stability through steric hindrance [15], independent of sugar and calcium ions [16,17], making them ideal next-generation stabilisers. Furthermore, extracting such high-value pectins [18] from underutilized agricultural by-products has emerged as a sustainable and economically viable strategy. Therefore, isolating and characterizing novel RG-I-type pectins from sources such as Muzao [19,20] offers a promising opportunity to both upgrade agricultural waste and meet market demand.

Pectin is commonly an acidic, polar macromolecule due to its high GalA and GlcA content [21]. In the context of fruit juice stabilisation, where the pH is typically maintained around 4.0, a highly branched pectin fraction might exhibit superior solubility and interaction properties [22]. However, such fractions from specific sources like Muzao remain largely unexplored. This knowledge gap limits the targeted development of Muzao-based functional ingredients. Therefore, we focused on isolating such a fraction from Muzao. The highly branched polysaccharide (ZMP2) was eluted with 0.2 M NaCl from ion exchange chromatography. After purification, its primary structure was well-characterised using a combination of monosaccharide composition analysis, FT-IR spectroscopy, methylation analysis, and NMR spectroscopy [23,24,25]. This study aims to characterize the fine structure of a highly branched pectin (ZMP2) isolated from Muzao fruit to evaluate its potential as a novel natural stabiliser for beverages.

## 2. Results

### 2.1. Isolation of the Weak Acidic

The jujube is rich in pectin, a good stabiliser and thickener, and has potential applications in fruit juice production [26]. As we all know, the pH of fruit juice always ranges 4.0~4.5, maintaining the sweet and sour taste [27]. Pectin contains a high proportion of GalA and GlcA, which are acidic polysaccharides [28]. But we should select the weekly acidic pectin as the stabiliser and thickener for fruit juice production.

**Figure 1:**
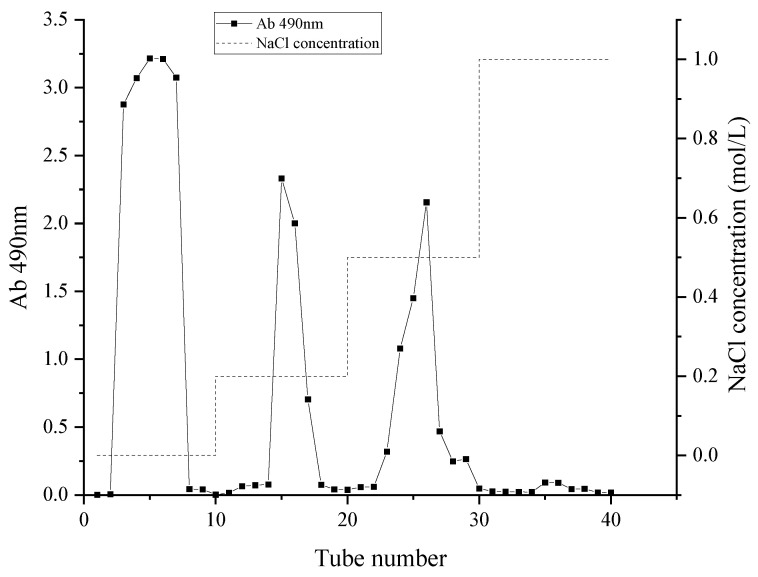
The DEAE-52 ion-exchange chromatography profile of ZMP.

The crude polysaccharide was extracted from Muzao (ZMP) with a yield of 2.34 ± 0.03% (*w*/*w*). After preparation, DEAE-52 ion-exchange chromatography has been employed to isolate ZMP. During the isolation, four fractions have been collected, eluted with water, 0.2 M NaCl, 0.5 M NaCl, and 1.0 M NaCl, corresponding to neutral, weakly acidic, acidic, and strongly acidic polysaccharides, respectively. The yields of each fraction are 48%, 7.4%, 15%, and 2% (Figure 1). Among them, we have collected the weakly acidic one (20~40 min) for further structure studies.

### 2.2. Purification of the ZMP2

**Figure 2:**
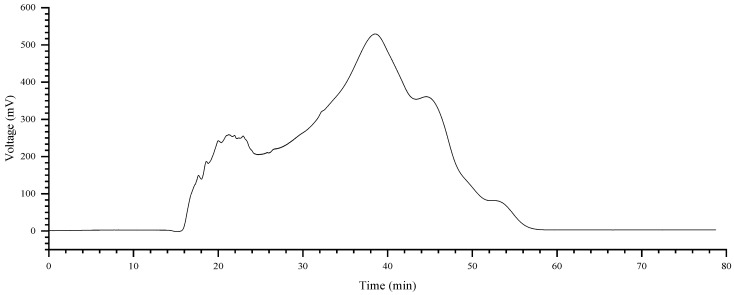
The weakly acidic polysaccharide chromatogram profile.

Gel chromatography is a critical step for polysaccharide purification, capable of separating polysaccharides with different degrees of polymerization [29,30]. In this study, the weakly acidic polysaccharide fraction, previously obtained by DEAE-52 chromatography, was further purified using a new automated gel purification system (BRT-GS) equipped with a differential refractive index detector (RID). As shown in the chromatogram (Figure 2), a single, prominent peak eluted between 30 and 45 min. This symmetrical peak was collected, dialyzed, concentrated, and freeze-dried to obtain the final purified fraction, designated as ZMP2.

The homogeneity of ZMP2 was then rigorously assessed. The HPGPC profile of ZMP2 revealed a single, sharp, and symmetrical peak, indicating that it is a homogeneous polysaccharide in terms of its molecular weight distribution. However, the total carbohydrate content of ZMP2, determined by the phenol-sulfuric acid method, was 55.32 ± 0.23%. This suggests that while ZMP2 is molecularly homogeneous, it contains a significant amount of non-carbohydrate impurities, most likely residual salts and small molecules from the purification buffers, which were not completely removed by dialysis. Despite this, the high molecular weight homogeneity makes ZMP2 suitable for subsequent structural and activity studies. To further investigate its structural features, ZMP2 was subjected to partial acid hydrolysis to generate smaller fragments. Specifically, ZMP2n5 and ZMP2y5 were prepared by hydrolyzing ZMP2 with 5 mL of 0.05 M TFA, while ZMP2n1 and ZMP2y1 were generated using 5 mL of 0.1 M TFA. The purity and molecular weight (Mw) of ZMP2 and its hydrolysates were then determined by HPGPC.

**Figure 3:**
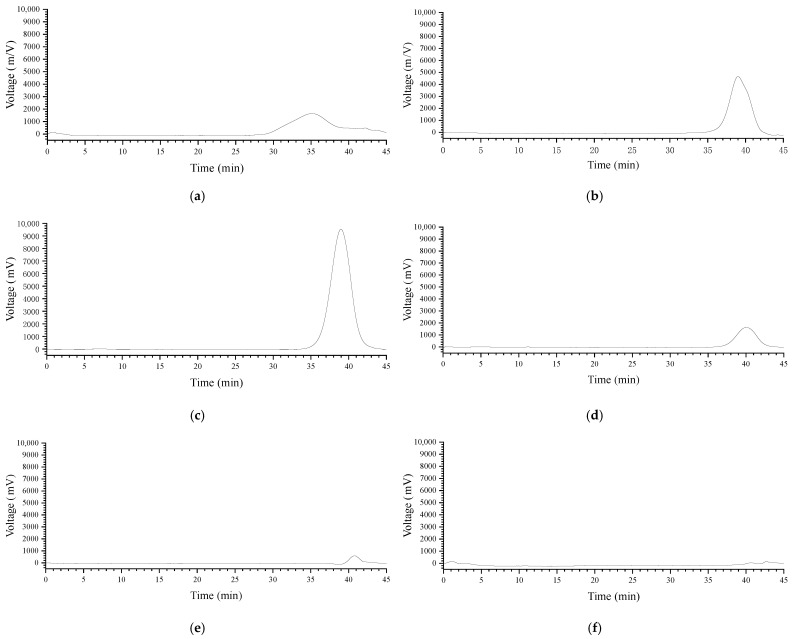
High-performance liquid chromatogram of each component of jujube polysaccharide. (**a**) ZMP; (**b**) ZMP2; (**c**) ZMP2n5; (**d**) ZMP2n1; (**e**) ZMP2y5; (**f**) ZMP2y1.

Figure 3 illustrates the purity and Mw of ZMP2, ZMP2n5, ZMP2y5, and ZMP2y1 by the HPGPC method. They all have a single and symmetrical peak. Their retention times were 39.01 min, 38.971 min, 40.064 min, and 40.763 min, respectively. According to the linear regression equation obtained from the standard curve of the molecular weight of polysaccharides, the Mw values of ZMP2, ZMP2n5, ZMP2n1, and ZMP2y5 can be calculated as 51,122 Da, 47,175 Da, 29,230Da, and 24,173 Da, respectively.

However, there is no prominent peak in the ZMP2y1 chromatogram, and further detection and analysis of the monosaccharide composition are required to assess the homogeneity of ZMP2.

### 2.3. Monosaccharides Composition

**Figure 4:**
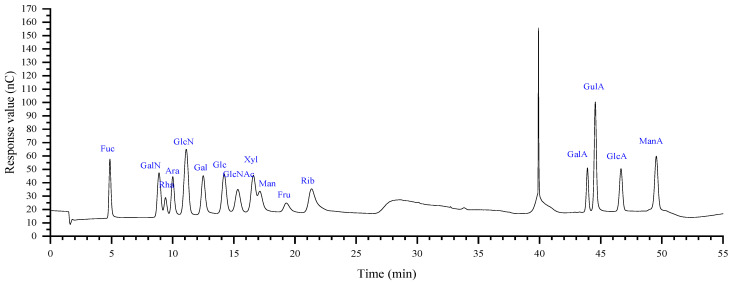
16 monosaccharide mixed standards in ion chromatogram (solvent peak: 2.0 min is the peak of sodium hydroxide, 40 min is the peak of sodium acetate).

Monosaccharide determination is the most fundamental indicator for preliminarily estimating polysaccharide species. Compared with other studies [31] that used a maximum of 12 monosaccharide standards, our study used 16 monosaccharide mixed standards (Figure 4).

Solvent peaks: The peak at 2.0 min corresponds to sodium hydroxide, and the peak at 40 min corresponds to sodium acetate.

Table 1 shows the monosaccharide composition of ZMP2 and its hydrolysis products (ZMP2n5, ZMP2y5, ZMP2n1, and ZMP2y1). They are all composed of rhamnose (Rha), arabinose (Ara), glucosamine hydrochloride (GlcN), galactose (Gal), glucose (Glc), xylose (Xyl), mannose (Man), and galacturonic acid (GalA). At the same time, Ara, Glc, and GalA are the main components. It is indicated that ZMP2 belongs to pectin, an acidic polysaccharide characterized by a high content of GalA and GlcA.

### 2.4. FT-IR Spectroscopy

**Figure 5:**
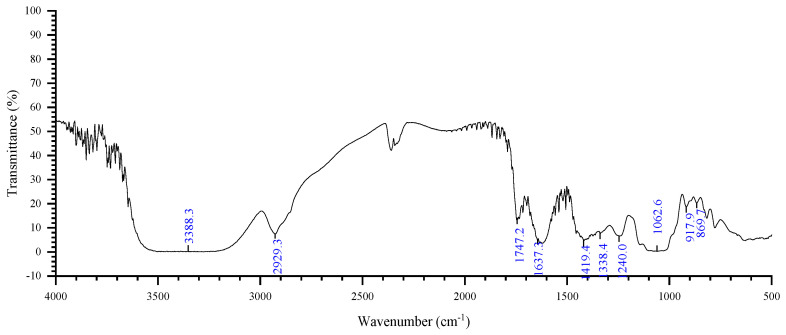
FT-IR spectrum of ZMP2.

FT-IR is a standard method for studying polysaccharide structure [32], in which different functional groups and chemical bonds are observed [33]. The FT-IR spectrum of ZMP2 is shown in Figure 5. The absorption band at 3600–3200 cm^−1^ is the stretching vibration absorption peak of –OH, and the absorption peak in this region is the characteristic peak of sugars. The details are as follows: 3388 cm^−1^ is the stretching vibration absorption peak of O–H, which is the characteristic peak of sugars [34]. There is an absorption peak at 2929 cm^−1^, which may be attributed to the C–H stretching vibration. There is an absorption peak at 1747 cm^−1^, which may be attributed to the C=O stretching vibration. There is an absorption peak at 1637 cm^−1^, which may be attributed to crystal water. There are absorption peaks at 1419 cm^−1^ and 1240 cm^−1^, which may be attributed to C–O stretching vibrations. There is an absorption peak at 1338 cm^−1^, which may be attributed to the C=O symmetric stretching vibration. There is an absorption peak at 1062 cm^−1^, which may be attributed to the O–H variable angle vibration. There is an absorption peak at 917 cm^−1^, which may be attributed to the asymmetric ring stretching vibration of the pyran ring. There is an absorption peak at 869 cm^−1^, which may be attributed to the rolling vibration of the cyclomethine of the pyran ring [35].

### 2.5. Glycosidic Linkage Patterns

The glycosidic linkage composition of a polysaccharide can be described by methylation analysis [36]. ZMP2 was hydrolysed, reduced, acetylated, and then determined by GC-MS. RXI-5 SIL MS column (30 m × 0.25 mm × 0.25 um) was used in GC-MS, and the programmed ramping conditions were set as below: starting temperature 120 °C, ramping at 3 °C/min to 250 °C/min; hold for 5 min; injector temperature 250 °C, detector temperature 250 °C/min, helium flow rate 1 mL/min.

Table 2 lists the distribution and ratios of methylated alditol acetates in ZMP2. Two main types of back bone linkage may be →4)-Galp-(1→ and →4)-Glcp-(1→ with a molar ratio of 0.216:0.232. The linkages →3,5)-Araf-(1→, →3,4)-Galp-(1→ and →3,6)-Galp-(1→ may indicate that ZMP2 is branched pectin. Methylation analysis is only a superficial method for pectin structure characterization, and NMR should be applied in further studies.

### 2.6. One-Dimensional NMR Analysis of ZMP2

**Figure 6:**
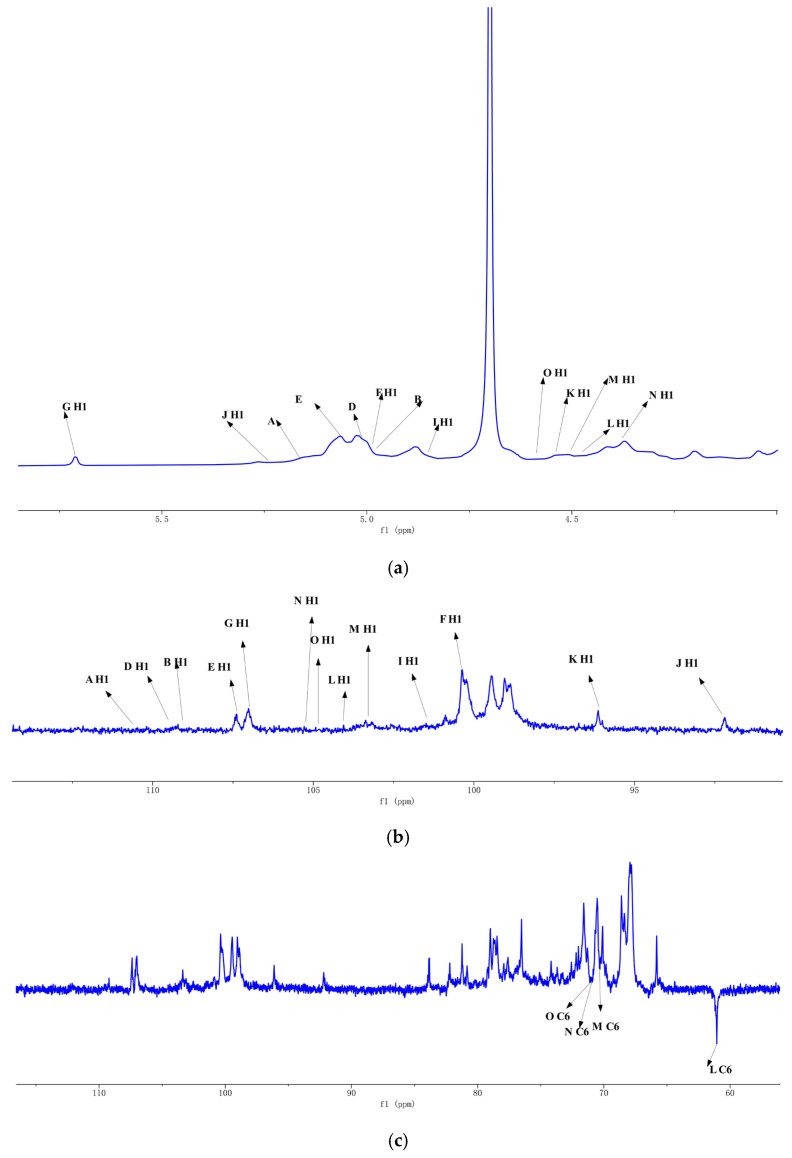
One-dimensional NMR spectra of ZMP2: (**a**) ^1^H spectrum, (**b**) ^13^C NMR spectrum, (**c**) ^13^C DEPT 135 spectrum.

1D NMR spectral analysis, including ^1^H NMR, ^13^C NMR, and DEPT 135, was performed to determine the structure of ZMP2. In ^1^H NMR spectra (Figure 6a), the peaks are mainly distributed between 3.0 and 5.5 ppm. The proton peaks for the sugar ring are in the range of 3.2–4.0 ppm, and the end-group proton peaks are concentrated in the region of 4.3~5.5 ppm, including prominent peaks at 4.39, 4.46, 4.5, 4.51, 4.55, 4.83, 4.96, 4.97, 5.01, 5.04, 5.17, 5.22, and 5.70 ppm.

^13^C NMR (201 MHz, D_2_O) spectrum (Figure 6b) shows that the significant signals are distributed between 60 and 120 ppm. The anomeric carbon region is mainly in the range of 93~180, and the major anomeric carbon signals are located at 93.62, 97.48, 100.38, 101.34, 103.83, 103.84, 104.3, 105.23, 108.18, 108.77, 108.88, 108.9, and 110.62 ppm. Other signals are distributed in 60~85 ppm region and the main signals are 69.6, 75.3, 79.12, 72.7, 72.82, 73.28, 79.19, 72.75, 69.4, 70.05, 79.15, 72.65, 72.7, 82.55, 80.28, 71.83, 76.7, 74.1, 77.25, 75.41, 70.2, 74.17, 71.58, 80.16, 76.69, 61.67, 72.79, 77.98, 70.05, 73.34, 70.6, 74.34, 76.18, 83.22, 74.1, 70.48, 72.1, 71.31, 70.7, 82.7, 77.8, 85.1, 62.33, 82.18, 78.12, 83.68, 68.27, 80.61, 83.65, 83.3, 67.8, 82.62, 77.97, 85.22, 62.64. Coanalyzed with the results of monosaccharide composition, ZMP2 mainly contains GalA, Gal, and Glc residues. The ^13^C DEPT 135 spectrum (Figure 6c) shows that the 61.67, 70.2, 70.48, and 70.6 ppm signals are C6 signals, where the peaks are inverted.

### 2.7. Two-Dimensional NMR Analysis of ZMP2

Further structural analysis of ZMP2 was conducted using 2D NMR spectra, including HH-COSY, HSQC, HMBC, and NOESY.

**Figure 7:**
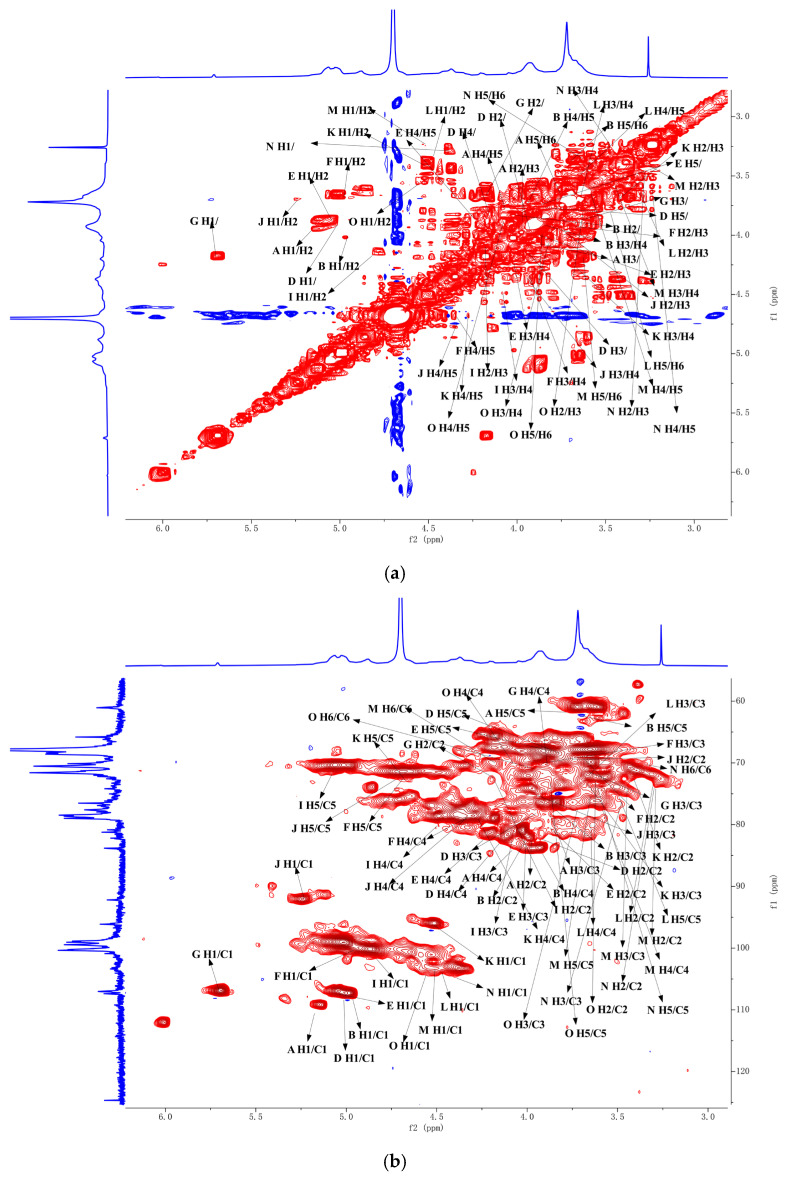

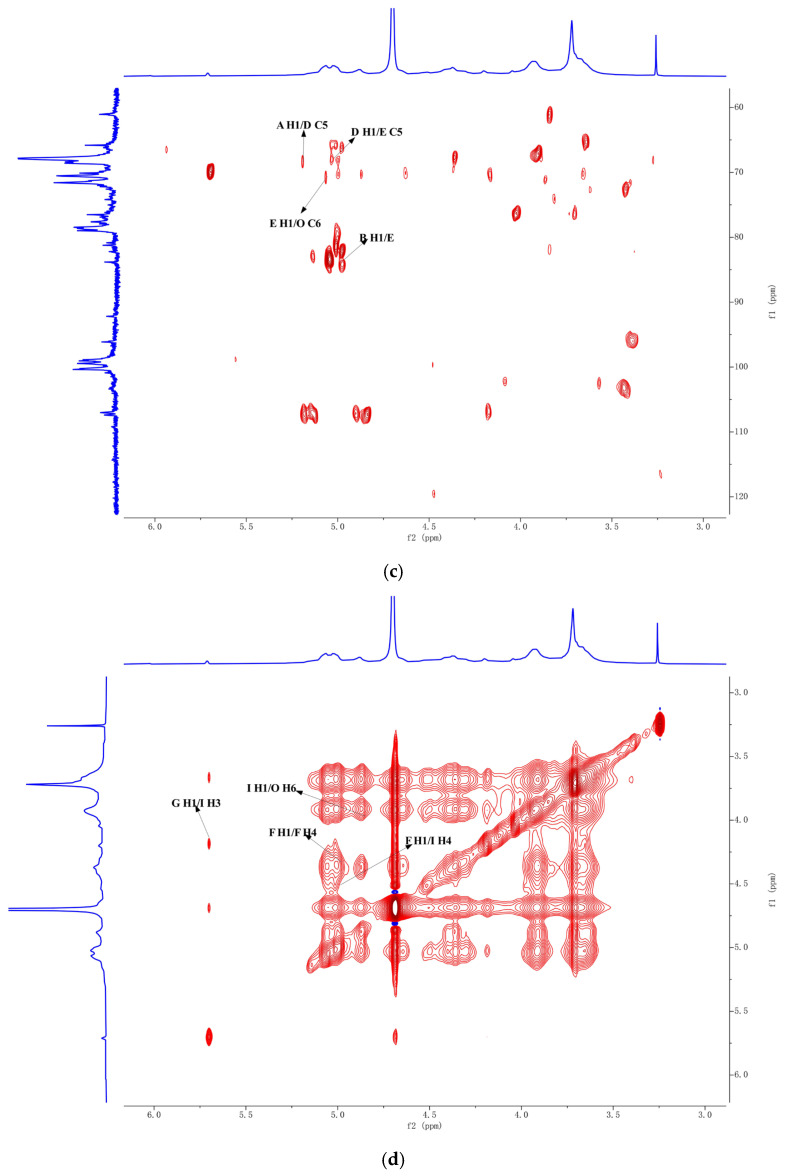
The two-dimensional NMR spectra of ZMP2: (**a**) ^1^H-^1^H-COSY spectrum, (**b**) HSQC spectrum, (**c**) HMBC spectrum, (**d**) NOESY spectrum. In the COSY and NOESY spectra, red contours represent positive cross-peaks, and blue contours represent negative cross-peaks. In the HSQC and HMBC spectra, red and blue contours indicate positive and negative intensities, respectively.

The HSQC spectrum (Figure 7b) shows an anomeric carbon signal at δ 100.38 and an anomeric hydrogen signal at δ4.97. In the 1H-1H-COSY spectrum (Figure 7a), the signals of H1–H2 are 4.97/3.67, H2–H3 are 3.67/3.93, H3–H4 are 3.93/4.32, and H4–H5a are 4.32/3.68. It can be deduced from the above that H1–H5 are respectively δ 4.97, 3.67, 3.93, 4.32, and 4.68, corresponding to C1–C5 with δ 100.38, 69.40, 70.05, 79.15, and 72.65, respectively. Therefore, the signal should be attributed to → 4)-α-GalpA-(1→.

Another anomeric carbon and hydrogen signal can be detected in the HSQC spectrum (Figure 7b) at δ 110.56 and 5.17, respectively. In the HH-COSY spectrum (Figure 7a), the signals of H1–H2 are 5.17/4.14, H2–H3 are 4.14/3.87, H3–H4 are 3.87/4.06, and H4–H5a are 4.06/3.76. It can be inferred that H1–H5a are δ5.17, 4.14, 3.87, and 3.76, and C1–C5 are 110.62, 82.62, 77.97, 85.22, and 62.64, respectively. Therefore, the signal should be α-L-Araf-(1→.

One more anomeric carbon and hydrogen signal can be detected, located at δ104.69 and δ4.47 in the HSQC spectrum (Figure 7b), respectively. In the 1H-1H-COSY spectrum (Figure 7a), the signals of H1–H2 are 4.47/3.57, H2–H3 are 3.57/3.68, H3–H4 are 3.68/4.05, H4–H5 are 4.05/3.87, and H5–H6a are 3.87/3.96. It can be deduced that the signals of H1–H6a are δ4.47, 3.57, 3.68, 4.05, 3.87, and 3.96, respectively. δ3.87, 3.96, and 104.69, 71.31, 81.5, 69.82, 74.81, and 70.76 for C1–C5, respectively; therefore, the signal should be →3,6)-Galp-(1→.

The complete ^1^H and ^13^C NMR signal assignments for residues are in Table 3.

The NOESY spectrum shows that the glycosidic bond →4)-α-D-GalAp-(1→ has a peak correlation between the anomeric hydrogen of the glycosidic bond and its own H4, suggesting the presence of a linkage mode of →4)-α-D-GalAp-(1→4)-α-D-GalAp-(1→.

In HMBC, the presence of a correlation peak between the anomeric hydrogen of the glycosidic bond →5)-α-L-Araf-(1→ and the C5 of its →3,5)-α-L-Araf-(1→ indicates the presence of →5)-α-L-Araf-(1→3,5)-α-L-Araf-(1→. Similarly, the presence of a correlation peak between the anomeric hydrogen of →3,5)-α-L-Araf-(1→ and the C6 of →3,6)-β-D-Galp-(1→ indicates the presence of →3,5)-α-L-Araf-(1→3,6)-β-D-Galp-(1→.

The anomeric hydrogen of →4)-α-D-GalAp-(1→ and the H4 of →3,4)-α-D-GalAp-(1→ has a correlation peak indicating the presence of →4)-α-D-GalAp-(1→3,4)-α-D-GalAp-(1→. The anomeric hydrogen of →3,4)-α-D-GalAp-(1→ has a correlation peak with H6 of →3,6)-β-D-Galp-(1→, indicating the presence of a glycosidic bond →3,4)-α-D-GalAp-(1→3,6)-β-D-Galp-(1→.

**Figure 8:**
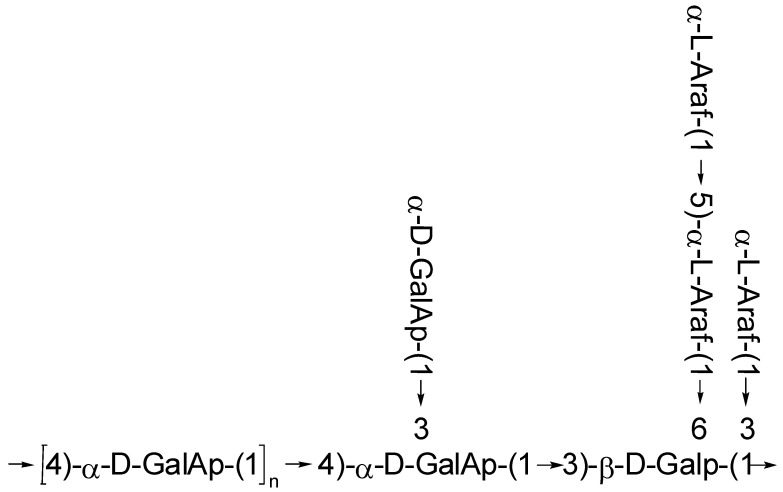
Structural Formula of ZMP2.

In summary, the primary structure of ZMP2 should be as follows: the main chain is connected as →4)-α-D-GalAp-(1→3)-β-D-Galp-(1→, while the end glycosidic bonds of α-D-Galp-(1→ and α-L-Araf-(1→5)-α-L-Araf-(1→ are attached to the main chain by O-3 and O-6 bonds from →3,4)-α-D-GalAp-(1→ and →3,6)-β-D-GalAp-(1→, respectively. The structural formula is shown below as Figure 8:

## 3. Discussion

The successful isolation and structural elucidation of ZMP2, a homogeneous polysaccharide from *Ziziphus jujuba* cv. *Muzao*, provides significant insights into the potential of underutilized botanical sources as functional food ingredients. Our findings move beyond simple characterization to establish a compelling structure–function rationale for its application as a natural stabiliser in weakly acidic environments, such as fruit and vegetable juices. The core of our argument rests on the identification of ZMP2 as a highly branched Rhamnogalacturonan-I (RG-I) pectin, a structural class whose unique properties are increasingly recognized for superior functional performance compared to conventional pectin sources.

The structural evidence robustly supports this classification. The high molar content of galacturonic and glucuronic acids (0.479 mol% and 0.071 mol%, respectively) confirms its identity as a pectic polysaccharide. This is further corroborated by spectroscopic signatures, including the characteristic ester carbonyl absorption near 1740 cm^−1^ in the FT-IR spectrum and anomeric carbon signals at approximately 100 ppm in the ^13^C NMR spectrum. Critically, the fine primary structure reveals a distinctive “backbone-side chain” architecture. The main chain, composed of alternating →4)-α-D-GalAp-(1→ and →3)-β-D-Galp-(1→ residues, is extensively decorated with arabinan and galactan side chains via O-3 and O-6 linkages. This highly branched RG-I structure is not merely a structural feature; we hypothesize that it is the origin of ZMP2’s functional potential. According to established principles of colloid science, such intricate side chains can create a thick hydration layer and provide significant steric hindrance [37], effectively preventing particle aggregation and conferring stability under acidic conditions. This mechanistic explanation, while requiring direct experimental validation, provides a strong theoretical foundation for our hypothesis.

When contextualized within the broader landscape of pectin research, the novelty of ZMP2 becomes apparent. Commercial pectins, predominantly derived from citrus peel and apple pomace, are rich in homogalacturonan (HG), often exceeding 80% of the polysaccharide content [38]. In stark contrast, ZMP2 exhibits the classic hallmarks of an RG-I pectin, aligning it more closely with bioactive polysaccharides isolated from medicinal plants like *Saposhnikovia divaricata* [39] and *Centella asiatica* [40]. This structural distinction is significant. A growing body of evidence suggests that RG-I pectins, due to their neutral sugar-rich side chains, offer superior steric stabilization and enhanced resistance to acid hydrolysis compared to their HG counterparts [41,42,43]. Therefore, the identification of a jujube-derived RG-I pectin not only expands our understanding of the structural diversity of *Ziziphus* polysaccharides but also positions ZMP2 as a promising candidate for applications where conventional HG pectins may fail.

Beyond its scientific novelty, the practical viability of this method must also be considered from an economic perspective. The extraction process relies on water as the primary solvent and utilizes standard chromatography techniques, making the procedure relatively simple and scalable. While the reported yield of ZMP2 is 2.34 ± 0.03% (*w*/*w*), the raw material, *Ziziphus jujube* cv. *Muzao*, is an abundant and low-cost agricultural byproduct [44]. This combination of an accessible feedstock and a straightforward purification protocol suggests that the method has a solid foundation for economic feasibility. Future optimization efforts, such as integrating membrane filtration [45,46] or exploring enzyme-assisted extraction [47,48,49], could further enhance the yield and reduce costs, strengthening its case for industrial-scale production.

This study, however, is not without limitations, which also delineate the path for future research. A primary constraint is that our conclusions on the stabilizing function of ZMP2 are inferred from its structure and have not yet been confirmed through direct functional assays in model beverage systems. Furthermore, our investigation was confined to the primary structure; the higher-order structures, such as molecular conformation, chain stiffness, and solution behavior under varying pH and ionic strength, remain unexplored. These factors are known to critically influence the functional properties of polysaccharides. Future work should therefore be directed towards experimentally validating the stabilizing performance of ZMP2 and systematically investigating its structure-activity relationship. Such studies will be essential for optimizing its extraction and purification processes, ultimately paving the way for its potential industrial application as a next-generation, clean-label food stabiliser.

## 4. Materials and Methods

### 4.1. Materials and Regents

*Zizyphus jujuba* cv. *Muzao* was purchased from Lin County, Lvliang City, Shanxi Province, China. NaCl and CF_3_COOH were purchased from ACROS (Fair Lawn, NJ, USA); 50% NaOH solution was purchased from Alfa Aesar (Ward Hill, MA, USA); CH_3_COONa and CH_3_COOH were purchased from Thermo Fisher (Waltham, MA, USA). Iodomethane (C_2_H_6_OS) and NaH were purchased from Adamas (Shanghai, China). NaBH4 and HClO_4_ were purchased from Aldrich (St. Louis, MO, USA). Methanol was purchased from Merck (Darmstadt, Germany). The above chemicals were of analytical grade.

Methylation kit, monosaccharide standards, and DEAE-52 cellulose were purchased from Borui Saccharide Co., Ltd. (Yangzhou, China); dextran standards 1152 were purchased from Shanghai Yuanye Biotechnology Co., Ltd. (Shanghai, China).

### 4.2. Crude Polysaccharide Preparation

The preparation of crude polysaccharides was adapted from established procedures [50,51,52] with minor modifications. Fresh Muzao fruits were washed, oven-dried at 60 °C to a constant weight, pitted, and crushed into a fine powder using a laboratory grinder. The powder (100 g) was mixed with distilled water at a material-to-liquid ratio of 1:10 (*w*/*v*). The extraction was performed at 100 °C for 3 h under constant stirring. This process was repeated three times in total. The combined supernatants were filtered through four layers of gauze and concentrated under reduced pressure at 60 °C using a rotary evaporator to approximately one-fifth of the original volume. Anhydrous ethanol was then added slowly to the concentrate with continuous stirring to a final ethanol concentration of 80% (*v*/*v*). The mixture was left to precipitate at 4 °C overnight. The precipitate was collected by filtration, redissolved in a minimal volume of distilled water, and deproteinized using the Sevag method (chloroform: n-butanol, 4:1, *v*/*v*). The mixture was shaken vigorously for 5 min and then centrifuged at 5000× *g* for 10 min at 4 °C. This deproteinization step was repeated until no white interface was observed. The resulting aqueous phase was transferred to a dialysis bag (molecular weight cutoff: 1000 Da) and dialyzed against running tap water for 48 h, followed by distilled water for an additional 24 h. The dialysate was finally concentrated and freeze-dried to obtain the crude ZMP.

### 4.3. DEAE-52 Ion-Exchange Chromatography

The purification procedure was adapted from a previously reported method [53] with modifications. DEAE-52 cellulose (100 g, dry weight) was pretreated and packed into a glass column (5 cm × 60 cm, I.D. × H), resulting in a column volume (CV) of approximately 1178 mL. The resin was first washed with distilled water until the effluent reached neutral pH (approximately 5 CV). The column was then equilibrated with distilled water at a flow rate of 5 mL/min for 2 h. Crude ZMP (2.0 g) was dissolved in 50 mL of distilled water, heated to 60 °C to ensure complete dissolution, and then centrifuged at 8000× *g* for 10 min to remove insoluble impurities. The clear supernatant was carefully loaded onto the equilibrated column. The column was eluted with a stepwise gradient of NaCl solutions at a flow rate of 15 mL/min: (1) distilled water (0–3 CV), (2) 0.2 M NaCl (3–6 CV), (3) 0.5 M NaCl (6–9 CV), and (4) 1.0 M NaCl (9–12 CV). Eluates were collected in 5 mL tubes. The carbohydrate content in each tube was monitored offline using the phenol-sulfuric acid method, measuring the absorbance at 490 nm. An elution curve was plotted by plotting absorbance versus elution volume. Based on the elution curve, fractions corresponding to distinct peaks were pooled. The target fraction, eluted with 0.2 M NaCl (designated as ZMP2), was concentrated, dialyzed against distilled water using a 3500 Da molecular weight cutoff membrane, and subsequently freeze-dried for further analysis.

### 4.4. Purification

The fraction eluted with 0.2 M NaCl from the ion-exchange step was further purified using gel filtration chromatography, following a procedure adapted from the literature [54] with specific modifications. The target fraction was dissolved in the mobile phase (0.05 M NaCl) to a suitable concentration and centrifuged at 12,000 rpm for 10 min. The resulting supernatant was loaded onto a fully automated gel purification system (BRT-GS). The purification process was monitored online using a differential refractive index detector (RI-502, SHODEX). Based on the real-time chromatogram, the main symmetrical peak was automatically collected. The collected fractions were pooled, dialyzed against distilled water using a 3500 Da molecular weight cutoff membrane, concentrated using a rotary evaporator, and subsequently freeze-dried to obtain the purified polysaccharide, designated as ZMP2.

### 4.5. Purity and Molecular Weight Determination by HPGPC

The purity and molecular weight (Mw) of the crude polysaccharide (ZMP), the purified fraction (ZMP2), and the partially hydrolyzed fractions (see Section 4.6) were determined by High-Performance Gel Permeation Chromatography (HPGPC) [55]. The samples were prepared as 5 mg/mL solutions, centrifuged at 12,000 rpm for 10 min, and the supernatants were filtered through 0.22 μm membranes. The HPGPC analysis was performed on a BRT105–104-102 series gel column (8 mm × 300 mm) maintained at 40 °C. The mobile phase was 0.05 M NaCl solution, delivered at a flow rate of 0.6 mL/min. A 20 μL aliquot of the filtered sample solution was injected for each run. Elution was monitored using a differential refractive index detector (RI-10A). A series of dextran standards with known molecular weights were used to generate the calibration curve (lg Mw = a + bRT, where R^2^ > 0.99). The Mw of the samples was calculated from their retention times based on this calibration equation.

### 4.6. Preparation of Partially Hydrolyzed Fractions

To investigate the structural features of ZMP2, a partial acid hydrolysis was performed. Briefly, 100 mg of ZMP2 was hydrolyzed with 5 mL of 0.05 M trifluoroacetic acid (TFA) at 80 °C for 1 h. The reaction was terminated by neutralizing the mixture to pH 7.0 with 1 M NaOH. The hydrolysate was then placed in a 3500 Da dialysis bag and dialyzed against distilled water for 24 h, with the water changed every 6 h. This yielded two fractions: the retentate (ZMP2n5, >3.5 kDa) and the permeate (ZMP2y5, <3.5 kDa). A second hydrolysis was conducted on the retentate (ZMP2n5) using 5 mL of 0.1 M TFA under the same conditions. Following neutralization and dialysis as described above, two additional fractions were obtained: ZMP2n1 (>3.5 kDa) and ZMP2y1 (<3.5 kDa). The molecular weight distributions of these hydrolyzed fractions were also analyzed by HPGPC under the same chromatographic conditions as described in Section 4.5.

### 4.7. Monosaccharides Composition Determination

The monosaccharide composition of ZMP, ZMP2, ZMP2n5, ZMP2y5, ZMP2n1, and ZMP2y1 was determined using ion chromatography (IC), following a method adapted from the literature [56] with modifications.

#### 4.7.1. Preparation of Standards and Samples

A mixed standard solution was prepared by accurately diluting individual stock solutions of 16 monosaccharide standards (fucose, rhamnose, arabinose, galactose, glucose, xylose, mannose, fructose, ribose, galacturonic acid, glucuronic acid, galactosamine hydrochloride, glucosamine hydrochloride, N-acetyl-D-glucosamine, guluronic acid, and mannuronic acid) to a series of known concentrations. Quantification was performed using an absolute quantification method, where the mass of each monosaccharide was measured and subsequently converted to a molar ratio based on its molecular weight.

For sample preparation, 10 mg of each polysaccharide sample was accurately weighed into a sealed ampoule. Then, 10 mL of 3 M TFA was added, and the hydrolysis was carried out at 120 °C for 3 h. After cooling, the hydrolysate was accurately transferred to a tube and evaporated to dryness under a stream of nitrogen. The residue was reconstituted in 10 mL of distilled water and vortexed to mix. An aliquot of 100 μL was taken, diluted with 900 μL of deionized water, and centrifuged at 12,000 rpm for 5 min. The resulting supernatant was directly injected for IC analysis.

#### 4.7.2. Ion Chromatography Conditions

The IC analysis was performed on a system equipped with a Dionex Carbopac™ PA20 analytical column (3 mm × 150 mm). The column temperature was maintained at 30 °C. The mobile phase consisted of (A) H_2_O, (B) 15 mM NaOH, and (C) 15 mM NaOH & 100 mM NaOAc. The flow rate was 0.3 mL/min, and the injection volume was 5 µL. The eluted carbohydrates were detected using an electrochemical detector.

### 4.8. Fourier Transform Infrared Spectrometer (FT-IR)

The functional groups of the purified polysaccharide ZMP2 were characterized using Fourier Transform Infrared (FT-IR) spectroscopy. Approximately 2 mg of ZMP2 was thoroughly mixed with 200 mg of dry, spectroscopic-grade potassium bromide (KBr) powder. The mixture was ground into a fine homogenous powder and then pressed into a transparent pellet under high pressure. A blank KBr pellet, prepared without any sample, served as the background reference. The FT-IR spectrum was recorded in the wavenumber range of 4000 to 400 cm^−1^ with a resolution of 4 cm^−1^. For each sample, 32 scans were accumulated and averaged to obtain a high signal-to-noise ratio. The resulting spectrum was analyzed to identify the characteristic functional groups present in ZMP2.

### 4.9. Methylation Analysis

The glycosidic linkages in ZMP2 were determined by methylation analysis, followed by GC-MS, according to a previously reported method [57,58] with modifications. The procedure consisted of three main steps: methylation, hydrolysis and derivatization, and GC-MS analysis.

#### 4.9.1. Methylation

Approximately 2–3 mg of dried ZMP2 was dissolved in 1 mL of anhydrous dimethyl sulfoxide (DMSO) in a sealed glass reaction flask. Methylation reagent A was added quickly, and the mixture was sonicated to dissolve. Subsequently, methylation reagent B was added. The reaction was carried out in a magnetic stirring water bath at 30 °C for 60 min. Finally, 2 mL of ultrapure water was added to terminate the methylation reaction. The methylation procedure was repeated two more times to ensure complete methylation, which was confirmed by the disappearance of the O-H absorption band in the FT-IR spectrum of the methylated product.

#### 4.9.2. Hydrolysis, Reduction, and Acetylation

The fully methylated polysaccharide was hydrolyzed with 1 mL of 2 M TFA for 90 min. The TFA was removed by co-evaporation to dryness. The residue was then reduced with 60 mg of sodium borohydride (NaBH_4_) in 2 mL of double-distilled water for 8 h. The reaction was neutralized by adding glacial acetic acid, concentrated under reduced pressure, and dried in an oven at 101 °C. The dried sample was then acetylated with 1 mL of acetic anhydride at 100 °C for 1 h. After cooling, 3 mL of toluene was added, and the mixture was concentrated and evaporated under reduced pressure. This procedure was repeated 4–5 times to remove excess acetic anhydride. The resulting partially methylated alditol acetates (PMAAs) were dissolved in 3 mL of CH_2_Cl_2_ and transferred to a separatory funnel. A small amount of distilled water was added, and after vigorous shaking, the upper aqueous layer was removed. This washing process was repeated 4 times. The CH_2_Cl_2_ layer was dried over an appropriate amount of anhydrous sodium sulfate, filtered, and the final volume was adjusted to 10 mL in a liquid-phase vial for GC-MS analysis.

#### 4.9.3. GC-MS Analysis

The PMAAs were analyzed on a Shimadzu GCMS-QP 2010 system equipped with an RXI-5 SIL MS capillary column (30 m × 0.25 mm i.d., 0.25 μm film thickness). The column temperature was programmed as follows: the initial temperature was 120 °C, then it was increased to 250 °C at a rate of 3 °C/min, and held at 250 °C for 5 min. Helium was used as the carrier gas at a flow rate of 1.0 mL/min. The injector temperature was 250 °C, and the detector temperature was 250 °C. The mass spectrometer was operated in electron impact (EI) mode at 70 eV, with a scan range of *m*/*z* 50–600. The PMAAs were identified by comparing their retention times and mass spectra with those in the NIST/Wiley mass spectral library and confirmed by literature data.

### 4.10. Nuclear Magnetic Resonance (NMR) Analysis

The structural elucidation of ZMP2 was performed using Nuclear Magnetic Resonance (NMR) spectroscopy. Approximately 50 mg of ZMP2 was dissolved in 0.5 mL of D_2_O (99.9%) and lyophilized. This dissolution-lyophilization cycle was repeated three times using fresh D_2_O to fully exchange the protons. The final sample was dissolved in 0.5 mL of D_2_O, and a small amount of deuterated acetone was added as an internal reference. The solution was then transferred to a 5 mm NMR tube. All NMR experiments were carried out on a Bruker 600 MHz spectrometer at 25 °C.

One-dimensional spectra, including ^1^H NMR, ^13^C NMR, and DEPT-135, were acquired. For the ^1^H NMR spectrum, the spectral width was 20 ppm, and the relaxation delay (D1) was 1.0 s. For the ^13^C NMR spectrum, the spectral width was 240 ppm, and the relaxation delay was 2.0 s.

Two-dimensional experiments, including ^1^H-^1^H COSY, HSQC, HMBC, and NOESY, were also performed to assign the signals. The HSQC experiment was optimized for a one-bond ^1^J_CH coupling constant of 145 Hz. The HMBC experiment had a long-range coupling delay of 80 ms. The NOESY mixing time was set to 300 ms.

### 4.11. Statistical Analysis

All experiments were performed in at least three independent replicates, and the data are presented as the mean ± standard deviation (SD). Statistical analyses were conducted using Origin 2021 software. Differences between two groups were evaluated using the Student’s *t*-test, while differences among multiple groups were assessed by one-way analysis of variance (ANOVA) followed by Tukey’s post hoc test. A *p*-value of less than 0.05 (*p* < 0.05) was considered to be statistically significant.

## 5. Conclusions

In this study, a homogeneous polysaccharide, designated ZMP2, was successfully isolated from *Ziziphus jujuba* cv. *Muzao*, and its fine primary structure was elucidated. Structural characterization revealed that ZMP2 is a highly branched RG-I-type pectin, with a distinctive main chain extensively decorated with arabinan and galactan side chains. Based on established structure–function relationships in polysaccharide science, this unique architecture suggests that ZMP2 possesses a high potential for application as a natural stabiliser in weakly acidic beverage systems. The elucidation of ZMP2’s structure enriches the structural library of jujube-derived polysaccharides and provides a novel substrate for future functional food research. Future work must prioritize the direct experimental validation of its hypothesized stabilizing properties and the systematic investigation of its structure-activity relationship to pave the way for potential industrial application.

## Figures and Tables

**Table 1:** Monosaccharide composition of ZMP2 and its hydrolysis products.

Monosaccharide	ZMP2 (mol%)	ZMP2n5 (mol%)	ZMP2y5 (mol%)	ZMP2n1 (mol%)	ZMP2y1 (mol%)
Fuc	0.000 ± 0.000	0.008 ± 0.001	0.015 ± 0.002	0.014 ± 0.003	0.000 ± 0.000
GalN	0.080 ± 0.005	0.048 ± 0.006	0.043 ± 0.004	0.053 ± 0.007	0.023 ± 0.002
Rha	0.000 ± 0.000	0.038 ± 0.005	0.018 ± 0.001	0.038 ± 0.004	0.039 ± 0.003
Ara	0.128 ± 0.010	0.185 ± 0.015	0.121 ± 0.011	0.174 ± 0.012	0.154 ± 0.013
GlcN	0.003 ± 0.001	0.002 ± 0.000	0.000 ± 0.000	0.002 ± 0.001	0.000 ± 0.000
Gal	0.097 ± 0.008	14.434 ± 0.821	0.116 ± 0.009	0.132 ± 0.010	0.103 ± 0.008
Glc	0.124 ± 0.009	16.334 ± 0.915	0.191 ± 0.012	0.098 ± 0.007	0.433 ± 0.025
GlcNAc	0.000 ± 0.000	0.000 ± 0.000	0.010 ± 0.001	0.002 ± 0.000	0.000 ± 0.000
Xyl	0.000 ± 0.000	18.925 ± 1.105	0.000 ± 0.000	0.015 ± 0.002	0.000 ± 0.000
Man	0.000 ± 0.000	19.542 ± 1.210	0.000 ± 0.000	0.012 ± 0.001	0.000 ± 0.000
Fru	0.000 ± 0.000	0.000 ± 0.000	0.000 ± 0.000	0.000 ± 0.000	0.000 ± 0.000
Rib	0.000 ± 0.000	0.000 ± 0.000	0.000 ± 0.000	0.000 ± 0.000	0.000 ± 0.000
GalA	0.497 ± 0.028	0.440 ± 0.032	0.443 ± 0.030	0.399 ± 0.025	0.191 ± 0.015
GulA	0.000 ± 0.000	0.000 ± 0.000	0.000 ± 0.000	0.000 ± 0.000	0.000 ± 0.000
GlcA	0.071 ± 0.006	0.062 ± 0.005	0.044 ± 0.003	0.055 ± 0.004	0.057 ± 0.004
ManA	0.000 ± 0.000	0.005 ± 0.001	0.000 ± 0.000	0.007 ± 0.001	0.000 ± 0.000

Note: Data are presented as mean ± standard deviation (SD, *n* = 3).

**Table 2:** Methylation analysis of ZMP2 and its hydrolysis products.

RT	Methylated Sugar	Mass Fragments (*m*/*z*)	Molar Ratio	Type of Linkage
17.74	2,3,5-Me3-Araf	43, 71, 87, 101, 117, 129, 145, 161	0.171 ± 0.012	Araf-(1→
20.788	2,4-Me2-Rhap	43, 58, 85, 89, 99, 117, 127, 131, 159, 201	0.055 ± 0.006	→3)-Rhap-(1→
21.365	2,4-Me2-Araf	43, 85, 99, 101, 117, 127, 161, 159	0.056 ± 0.005	→3)-Araf-(1→
23.596	2,3-Me2-Araf	43, 71, 87, 99, 101, 117, 129, 161, 189	0.058 ± 0.008	→5)-Araf-(1→
27.154	2,3,4,6-Me4-Galp	43, 71, 87, 101, 117, 129, 145, 161, 205	0.026 ± 0.003	Galp-(1→
27.966	2-Me1-Araf	43, 58, 85, 99, 117, 127, 159, 201	0.021 ± 0.004	→3,5)-Araf-(1→
29.817	2,3,6-Me3-Galp	43, 87, 99, 101, 113, 117, 129, 131, 161, 173, 233	0.216 ± 0.015	→4)-Galp-(1→
31.632	2,3,6-Me3-Glcp	43, 87, 99, 101, 113, 117, 129, 131, 161, 173, 233	0.232 ± 0.013	→4)-Glcp-(1→
34.784	2,6-Me2-Galp	43, 87, 99, 117, 129, 143, 159	0.051 ± 0.009	→3,4)-Galp-(1→
36.147	2,3-Me2-Galp	43, 71, 85, 87, 99, 101, 117, 127, 159, 161, 201, 261	0.053 ± 0.007	→4,6)-Galp-(1→
38.655	2,3-Me2-Glcp	43, 71, 85, 87, 99, 101, 117, 127, 159, 161, 201	0.030 ± 0.001	→4,6)-Glcp-(1→
39.813	2,4-Me2-Galp	43, 87, 117, 129, 159, 189, 233	0.032 ± 0.002	→3,6)-Galp-(1→

Note: Data are presented as mean ± standard deviation (SD, *n* = 3).

**Table 3:** ^1^H and ^13^C NMR chemical shifts of ZMP2.

Residues	H1/C1	H2/C2	H3/C3	H4/C4	H5/C5	H6a,b/C6	H6b
α-L-Araf-(1→	5.17	4.13	3.87	4.06	3.76	3.64	
	110.62	82.62	77.97	85.22	62.64		
α-L-Araf-(1→	4.96	4	3.83	3.91	3.69	3.36	
	108.77	82.7	77.8	85.1	62.33		
→5)-α-L-Araf-(1→	5.01	4.07	3.94	4.15	3.82	3.75	
	108.88	82.18	78.12	83.68	68.27		
→3,5)-α-L-Araf-(1→	5.04	4.21	4	4.24	3.86	3.75	
	108.9	80.61	83.65	83.3	67.8		
→4)-α-D-GalAp-(1→	4.97	3.67	3.93	4.32	4.68		3.73
	100.38	69.4	70.05	79.15	72.65	176.7	54.13
α-D-GalAp-(1→	5.7	4.17	3.64	3.87	ns		
	108.18	72.1	71.31	70.7	ns	176.5	
→3,4)-α-D-GalAp-(1→	4.83	4.17	4.09	4.48	5.07		
	101.34	72.7	82.55	80.28	71.83	176.7	
→4)-α-D-GalAp	5.22	3.74	3.95	4.34	4.72		
	93.62	69.6	75.3	79.12	72.7	172.36	
→4)-β-D-GalAp	4.51	3.41	3.66	4.27	4.66		
	97.48	72.82	73.28	79.19	72.75	172.16	
→4)-β-D-Glcp-(1→	4.46	3.3	3.63	3.64	3.47	3.95	3.79
	103.84	74.17	71.58	80.16	76.69	61.67	
→4,6)-β-D-Glcp-(1→	4.5	3.27	3.44	3.58	3.64	4.18	3.8
	103.83	76.7	74.1	77.25	75.41	70.2	
→4,6)-β-D-Galp-(1→	4.39	3.28	3.47	3.22	3.62	3.34	
	105.23	74.34	76.18	83.22	74.1	70.48	
→3,6)-β-D-Galp-(1→	4.55	3.62	3.81	4.08	3.87	3.96	3.65
	104.3	72.79	77.98	70.5	73.34	70.6	

Note: Chemical shifts are expressed in ppm relative to the internal standard (acetone-d6, δH 2.05, δC 31.5).

## Data Availability

The original contributions presented in the study are included in the article; further inquiries can be directed to the corresponding author.

## References

[1] Zhou P., Zhao L. (2020). Current situation and control measures of main diseases in Lyuliang jujube production area. Agric. Technol..

[2] Zhang B. (2025). Discussion on river levee treatment of Linxian section of Qiushui River. Shaanxi Water Resour..

[3] Ren Z. (2025). The 72 transformations of a small jujube. Shanxi Dly..

[4] Rahman M.J., Ambigaipalan P., Shahidi F. (2018). Biological activities of camelina and sophia seeds phenolics: Inhibition of LDL oxidation, DNA damage, and pancreatic lipase and α-glucosidase activities. J. Food Sci..

[5] Geng L., Shi Q., Zhou W., Wang D., Hang F., Wei H., Tertuliano M.I.E., Aabideen M.Z.U. (2024). Optimization of the extraction process of goji berry pectin using response surface methodology and its suitability as thickener for yogurt. Heliyon.

[6] Zhu Y., He Z., Bao X., Wang M., Yin S., Song L., Peng Q. (2021). Purification, in-depth structure analysis, and antioxidant stress activity of a novel pectin-type polysaccharide from *Ziziphus jujuba* cv. *Muzao* residue. J. Funct. Foods.

[7] Ferrer D.S., Wachtendorff D.L., Munizaga G.T., Won M.P. (2025). Effect of pulsed electric fields (PEF) and ultrasound (US) assisted-extraction technologies on the recovery of functional clean-label starches from banana peels. LWT.

[8] Masud A., Kirty P., Singh D.B., Nabi D.B., Vikas N. (2024). Exploring the versatility of diverse hydrocolloids to transform techno-functional, rheological, and nutritional attributes of food fillings. Food Hydrocoll..

[9] Contreras R.A., Nicholls A.M., Lauzan G., Vidal C., Sujanto M., Pizarro M. (2025). Screening and Optimization of Natural Hydrocolloids for the Stabilization of Pea Protein Solutions. Food Biophys..

[10] Ozturk O.K., Salgado A.M., Holding D.R., Campanella O.H., Hamaker B.R. (2023). Dispersion of zein into pea protein with alkaline agents imparts cohesive and viscoelastic properties for plant-based food analogues. Food Hydrocoll..

[11] Amorim T.A., Carvalho A.J.B.A., Figueiredo L.S., Lima M.S., Sarinho A.M., Santos N.C., Lisboa H.M., Gusmão T.A.S., Gusmão R.P. (2025). Structure-function relationship and antioxidant mechanisms of pectin from red and white pitaya peels for functional food applications. Food Hydrocoll..

[12] Kaur G., Khan Z.S., Toker Ö.S., Bhat M.S., Basyigit B., Kurt A., Rustagi S., Suri S., Hatami S., Fayaz S. (2024). Innovative approaches to pectin processing: Enhancing techno-functional properties for applications in food and beyond. Bioact. Carbohydr. Diet. Fibre.

[13] Khubber S., Kazemi M., Amiri Samani S., Lorenzo J.M., Simal Gandara J., Barba F.J. (2023). Structural-functional Variability in Pectin and Effect of Innovative Extraction Methods: An Integrated Analysis for Tailored Applications. Food Rev. Int..

[14] Condezo-Hoyos L., Cortés-Avendaño P., Lama-Quispe S., Calizaya-Milla Y.E., Méndez-Albiñana P., Villamiel M. (2024). Structural, chemical, and technofunctional properties of pectin modified by green and novel intermediate frequency ultrasound procedure. Ultrason. Sonochem..

[15] Zhang L., Vlach J., Black I.M., Archer Hartmann S., Heiss C., Azadi P., Urbanowicz B.R. (2025). The pectin puzzle: Decoding the fine structure of rhamnogalacturonan-I (RG-I) in Arabidopsis thaliana uncovers new pectin features. Carbohydr. Polym..

[16] Gu J., Zhao M., You L., Lin L. (2025). Demonstration of the effective intestinal immunity activity of a high-branched rhamnogalacturonan-I type pectin from wolfberry via exploration of its interaction with colon biological and mechanical barrier. Carbohydr. Polym..

[17] Xing Y., Chen G., Dong X., Kou L., Wang J., Xu H. (2025). Impact of high-pressure homogenization on the homogalacturonan and rhamnogalacturonan-I regions of pectin in strawberry pulp and their interaction with anthocyanins. Food Chem..

[18] Liu Y., Meng Y., Ji H., Guo J., Shi M., Lai F., Ji X. (2024). Structural characteristics and antioxidant activity of a low-molecular-weight jujube polysaccharide by ultrasound assisted metal-free Fenton reaction. Food Chem. X.

[19] Ji X., Hou C., Yan Y., Shi M., Liu Y. (2020). Comparison of structural characterization and antioxidant activity of polysaccharides from jujube (*Ziziphus jujuba* Mill.) fruit. Int. J. Biol. Macromol..

[20] Ji X., Yan Y., Hou C., Shi M., Liu Y. (2020). Structural characterization of a galacturonic acid-rich polysaccharide from *Ziziphus jujuba* cv. *Muzao*. Int. J. Biol. Macromol..

[21] Ji X., Zhang F., Zhang R., Liu F., Peng Q., Wang M. (2019). An acidic polysaccharide from *Ziziphus jujuba* cv. *Muzao*: Purification and structural characterization. Food Chem..

[22] Huo J., Zhang M., Sun Q., Chen Y. (2025). Combination of Hydrocolloid and Ultrasound Assistance to Improve the Stability of Lemon Juice After Thawing. J. Food Process. Eng..

[23] Ma S., Yang Z., Sun H., Wu T., Pan S., Xu X. (2025). Multiscale comparative study of pectin extraction from broccoli stalk using alkaline and enzymatic modifications: Structural characterization and gelling properties. Int. J. Biol. Macromol..

[24] Guo Y., Cheng Y., Zheng B., Xie J., Chen Y., Lin H., Liu H., Hu X., Yu Q. (2025). Structural characterization and immunomodulatory activity of a novel pectin polysaccharide extracted from *Gardenia jasminoides* fruit. Carbohydr. Polym..

[25] Fan R., Zhang W., Wang L., Fei T., Xiao J., Wang L. (2025). Structural Characterization of a Novel Pectin Polysaccharide from Mango (*Mangifera indica* L.) Peel and Its Regulatory Effects on the Gut Microbiota in High-Fat Diet-Induced Obese Mice. Foods.

[26] Lu X., Zhao C., Liu D., Hu M., Cui J., Wang F., Zeng L., Zheng J. (2024). A novel prebiotic enzymatic hydrolysate of citrus pectin during juice processing. Food Hydrocoll..

[27] Liviz C.A.M., Maciel G.M., Pedro A.C., Ribeiro I.S., Fernandes I.A.A., Pinheiro D.F., Lima N.P., Martins L.R.R., Haminiuk C.W.I. (2025). Physicochemical properties, bioactive compounds, and pesticide residues in commercial fruit juices. Microchem. J..

[28] Li Z., Zhang J., Zhang H., Liu Y., Zhu C. (2022). Effect of different processing methods of hawthorn on the properties and emulsification performance of hawthorn pectin. Carbohydr. Polym..

[29] Xie X., Shen W., Zhou Y., Ma L., Xu D., Ding J., He L., Shen B., Zhou C. (2020). Characterization of a polysaccharide from *Eupolyphaga sinensis* Walker and its effective antitumor activity via lymphocyte activation. Int. J. Biol. Macromol..

[30] Zhou Y., Qian C., Yang D., Tang C., Xu X., Liu E., Zhong J., Zhu L., Zhao Z. (2021). Purification, Structural Characterization and Immunomodulatory Effects of Polysaccharides from *Amomum villosum* Lour. on RAW 264.7 Macrophages. Molecules.

[31] Li J., Ai L., Hang F., Ding S., Liu Y. (2014). Composition and antioxidant activity of polysaccharides from jujuba by classical and ultrasound extraction. Int. J. Biol. Macromol..

[32] Zhou Y., Wang S., Feng W., Zhang Z., Li H. (2021). Structural characterization and immunomodulatory activities of two polysaccharides from Rehmanniae Radix Praeparata. Int. J. Biol. Macromol..

[33] Deng Y., Huang L., Zhang C., Xie P., Cheng J., Wang X., Liu L. (2020). Novel polysaccharide from Chaenomeles speciosa seeds: Structural characterization, α-amylase and α-glucosidase inhibitory activity evaluation. Int. J. Biol. Macromol..

[34] Luo D., Wang Z., Zhou R., Cao S. (2020). A polysaccharide from Umbilicaria yunnana: Structural characterization and anti-inflammation effects. Int. J. Biol. Macromol..

[35] Guo Q., Cui S.W., Kang J., Ding H., Wang Q., Wang C. (2015). Non-starch polysaccharides from American ginseng: Physicochemical investigation and structural characterization. Food Hydrocoll..

[36] Sims I.M., Carnachan S.M., Bell T.J., Hinkley S.F. (2018). Methylation analysis of polysaccharides: Technical advice. Carbohydr. Polym..

[37] Hu T., Tan F., Li L., An K., Zou B., Wen J., Wu J., Xiao G., Yu Y., Xu Y. (2023). Structural elucidation and physicochemical properties of litchi polysaccharide with the promoting effect on exopolysaccharide production by *Weissella confusa*. Int. J. Biol. Macromol..

[38] Méndez Albiñana P., Rodrigues Díez R., Rodríguez Rodríguez P., Moreno R., Muñoz Valverde D., Casani L., Blanco Rivero J. (2025). Structure and properties of citrus pectin as influencing factors of biomarkers of metabolic syndrome in rats fed with a high-fat diet. Curr. Res. Food Sci..

[39] Qi X., Liu Y., Zhou Y., Li H., Yang J., Liu S., He X., Li L., Zhang C., Yu H. (2025). A pectic polysaccharide from *Typhonii rhizoma*: Characterization and antiproliferative activity in K562 cells through regulating mitochondrial function and energy metabolism. Carbohydr. Polym..

[40] Xu X., He Z., Luo X., Peng J., Ning X., Mayo K.H., Tai G., Zhang M., Zhou Y. (2024). RG-I-containing sugar domains from *Centella asiatica* bind strongly to galectin-3 to inhibit cell–cell interactions. Chem. Biol. Technol. Agric..

[41] Huang G., Chen F., Yang W., Huang H. (2021). Preparation, deproteinization, and comparison of bioactive polysaccharides. Trends Food Sci. Technol..

[42] Wang Q., Xu J., Cheng H., Chen S., Ye X., Chen J. (2025). Galactan side chains dominate thermal aggregation of RGI-rich pectin during drying, resulting in poor solubility. Carbohydr. Polym..

[43] Mao Y., Dewi S.R., Harding S.E., Binner E. (2024). Influence of ripening stage on the microwave-assisted pectin extraction from banana peels: A feasibility study targeting both the Homogalacturonan and Rhamnogalacturonan-I region. Food Chem..

[44] Bai Y., Zhang H., Jia S., Sun D., Zhang J., Zhao X., Fang X., Wang X., Xu C., Cao R. (2024). Optimized sand tube irrigation combined with nitrogen application improves jujube yield as well as water and nitrogen use efficiencies in an arid desert region of Northwest China. Front. Plant Sci..

[45] Ippolitov V., Anugwom I., Mänttäri M., Mänttäri M.K. (2025). Comparison between membrane filtration and antisolvent addition methods for the recovery and recycling of a deep eutectic solvent after biomass pretreatment. Ind. Crops Prod..

[46] Wang T., Wu X., Li X., Feng W., Wang R., Huang K. (2025). Preparation of salt-free *Pleurotus eryngii* protein with enhanced colloidal stability and emulsifying properties by ceramic membrane filtration. Food Hydrocoll..

[47] Aung T., Nayab, Kim C.Y., Kim M.J. (2025). Optimized enzyme-assisted ultrasonic extraction and encapsulation of *Curcuma longa* for jelly incorporation and in-vitro bioactives release. Food Chem..

[48] Vo T.P., Nguyen T.H.T., Nguyen H.B.T., Nguyen H.N., Le N.V.N., Ha M.H., Pham G.B., Nguyen D.Q. (2025). Enhancing phenolic and flavonoid recovery from Vietnamese balm using green solvent-based ultrasonic-enzymatic-assisted extraction. Ultrason. Sonochem..

[49] Chen B.J., Khoo H.E., Li X., Peng B. (2025). Application of α-amylase-assisted extraction of longan seed polysaccharides in preparation of chromium-free tanning agents. Mater. Today Chem..

[50] Yang Y., Zheng Z., Gao L., Zhang Y., Zhang R. (2021). Ultrasound-assisted extraction, structural characterization and antioxidant activity evaluation of polysaccharides from Ziziphus jujuba. Food Ferment. Ind..

[51] Ji X., Yin M., Hou C., Liu Y. (2020). Research progress on extraction, separation, purification, and biological activities of *Ziziphus jujuba* polysaccharides. Sci. Technol. Food Ind..

[52] Zou X., Xiao J., Chi J., Zhang M., Zhang R., Jia X., Mei D., Dong L., Yang Y., Huang F. (2022). Physicochemical properties and prebiotic activities of polysaccharides from *Zizyphus jujube* based on different extraction techniques. Int. J. Biol. Macromol..

[53] Imiołek M., Fekete S., Rudaz S., Guillarme D. (2025). Ion exchange chromatography of biotherapeutics: Fundamental principles and advanced approaches. J. Chromatogr. A.

[54] Xie L., Cai W., Zhang H., Chen Y. (2012). Isolation, purification, and anticoagulant activity of tea polysaccharides. Food Ferment. Ind..

[55] Chen Q., Chen Y., Zhou S., Yip K.M., Xu J., Chen H., Zhao Z. (2018). Laser microdissection hyphenated with high-performance gel permeation chromatography-charged aerosol detector and ultra-performance liquid chromatography-triple quadrupole mass spectrometry for histochemical analysis of polysaccharides in herbal medicine: Ginseng, a case study. Int. J. Biol. Macromol..

[56] Huang X., He X., Yang Q., Zhang C., Zhou N. (2020). Determination of monosaccharide composition of Stachys sieboldii polysaccharides by PMP pre-column derivatization HPLC. Food Ferment. Ind..

[57] Nagar S., Lakhera A.K., Kumar V. (2020). Upgrading Methylation Method for Structural Studies of Polysaccharides: Case Analysis of a Bioactive Polysaccharide from Acacia tortilis. J. Biol. Act. Prod. Nat..

[58] Li Y., Gao J.N., Liang J., Zhu X.H., Kuang H.X., Xia Y.G. (2025). An alternative GC–MS library for methylation analysis of polysaccharides via partially methylated aldononitrile acetates. Carbohydr. Polym..

